# Characterization, Physical Properties, and Biocompatibility of Novel Tricalcium Silicate–Chitosan Endodontic Sealer

**DOI:** 10.1055/s-0042-1745774

**Published:** 2022-09-05

**Authors:** Ike D. Maharti, Endang Suprastiwi, Harry Agusnar, Nendar Herdianto, Anggraini Margono

**Affiliations:** 1 Doctoral Program, Faculty of Dentistry, Universitas Indonesia, Jakarta, Indonesia; 2 Department of Conservative Dentistry, Universitas Indonesia, Jakarta, Indonesia; 3 Department of Chemistry, Faculty of Mathematics and Natural Science, Universitas Sumatera Utara, Medan, Indonesia; 4 Research Center for Advanced Materials (PRMM-BRIN), Banten, Indonesia

**Keywords:** biocompatibility, chitosan, cytotoxicity, physicochemical, root canal sealer, tricalcium silicate

## Abstract

**Objective** The purpose of this study was to compare the characteristics, physical properties, and biocompatibility of the novel tricalcium silicate–chitosan (TCS-C) sealer with AH Plus and Sure-Seal Root.

**Materials and Methods** The TCS-C powder was prepared by mixing tricalcium silicate with 2% water-soluble chitosan at a 5:1 ratio, followed by sufficient addition of 10 g/mL ratio of double-distilled water to form a homogeneous cement. Material characterizations (the Fourier Transform InfraRed [FTIR] and X-ray diffraction [XRD]), physical property investigations (flow and film thickness), and cytotoxicity tests in 3T3 mouse embryo fibroblast cell (MTT assay method) were performed on sealers, and the results were compared with those of the commercial products.

**Statistical Analysis** Statistical analysis was performed on flow and film thickness. The normality of the data was tested using the Shapiro–Wilk test. Statistical analysis was performed with one-way analysis of variance (ANOVA). The level of significance was set at *p* < 0.05.

**Results** The TCS-C showed a mean flow of 31.98 ± 0.68 mm, compared with Sure Seal Root at 26.38 ± 0.69 mm and AH Plus at 26.50 ± 0.12 mm. The TCS-C showed a mean film thickness of 60 ± 10.0 mm compared with Sure-Seal Root at 50 ± 10.0 mm and AH Plus at 40 ± 15.8 mm. The TCS-C exhibited low to no cytotoxicity in fibroblast cell at all concentrations and exposure times.

**Conclusion** Adding water-soluble chitosan may improve the physical and biologic properties of tricalcium silicate cement. The novel TCS-C sealer did not fully meet the physical properties of an endodontic sealer, but it was not cytotoxic to fibroblast cells.

## Introduction

Endodontic sealers are used for the obturation of root canal systems to achieve a fluid-tight seal between the dentinal wall and the core filling material throughout the entire canal. A root canal sealer must demonstrate appropriate physicochemical and biological properties. Grossman stated that an ideal root canal sealer should possess excellent sealing ability, dimensional stability, a slow setting time, insolubility, and biocompatibility. 1 Currently, various sealers have been developed for endodontic treatment. The bioceramic sealer is the latest generation of sealers and consists mainly of calcium silicate which is claimed to have benefits in biocompatibility, bioactivity, and osteoconductivity and to apparently favor the regeneration of apical tissues. 2 3 4 Despite its advantages in biocompatibility, poor handling properties still remain to be resolved. 2 4 5 It is known to have long setting time, low flow ability, and high film thickness.

At present, no ideal sealers are available for endodontic treatment. For this reason, dental material industries are now competing to create and to modify calcium silicate–based sealers because of their promising properties for the success of root canal treatments and tissue healing. Chitosan, a natural biopolymer, is a suitable biomaterial for various clinical applications, as its benefits include high biocompatibility, low elicitation of inflammatory responses, antibacterial activity, and high biodegradability. It can be used as a temporary scaffolding support for tissue growth and regeneration. 6 7 However, chitosan has two major limitations: it is not soluble in water and it has a low pH. The chitosan formulation used in this study was chitosan derivative (water-soluble chitosan) with lower molecular weight and has been developed to dissolve in water. 8 This lower molecular weight means lower viscosity and it is believed to be easily bind to calcium silicate particles providing density and strength of its structure. Flow ability of sealer depends on its viscosity and low viscosity will provide higher flow ability.

Besides its excellent biological properties, its easy manipulation making it a suitable component for use in hydrogels. 5 Adding chitosan in calcium silicate–based cement may improve its handling properties. 5 9 10 Therefore, in the present study, a novel bioceramic-chitosan sealer was considered a better candidate for endodontic biomaterial due to its excellent physicochemical and biological properties. 11 The null hypothesis was that the incorporation of water-soluble chitosan and tricalcium silicate cement creates an endodontic sealer material that has good flow ability, film thickness, and biocompatibility on fibroblast cell. However, little information is available regarding incorporation water-soluble chitosan and tricalcium silicate characteristics as endodontic sealers. The purpose of this study was to compare the characteristics, physical properties, and biocompatibility of the novel tricalcium silicate–chitosan (TCS-C) sealer with AH Plus (an epoxy-resin-based gold-standard sealer) and Sure-Seal Root (a calcium silicate-based sealer).

## Materials and Methods

This study was approved by the Commission of Ethical Research in Dentistry, Faculty of Dentistry, Universitas Indonesia, number: 49/Ethical Approval /FKGUI/X/2020 with protocol number: 070260820. The novel TCS-C sealer was prepared from water-soluble chitosan (PUI Kitosan dan Material Maju, Universitas Sumatera Utara, Medan, Indonesia) and tricalcium silicate powder. The tricalcium silicate powder was synthesized from calcium silicate (Sigma-Aldrich, Singapore), tricalcium phosphate (Merck, Indonesia), and calcium hydroxide (Merck, Indonesia). The tricalcium silicate powder was manually mixed with 2% water-soluble chitosan at a 5:1 ratio, followed by the addition of sufficient double-distilled water at a 10 g/mL ratio until a homogenous cement was obtained. AH Plus and Sure-Seal Root were mixed according to the manufacturers' instructions.

The material characterization tests included the Fourier Transform InfraRed (FTIR) spectroscopy and X-ray diffraction (XRD). The physical property tests included flow and film thickness determinations according to ISO 6876/2012. The biocompatibility was tested on the 3T3 mouse embryo fibroblast cell line using the MTT assay method. 12 The data were analyzed and compared with the data obtained for AH Plus (Dentsply Sirona, Charlotte, USA), Sure-Seal Root (Sure Dent Corporation, Gyeonggi-do, South Korea), and the ISO 6876/2012 standards.

### Assessment of the Fourier Transform InfraRed and X-Ray Diffraction

Each sealer was mixed and poured into a mold with a diameter of 10 mm and a thickness of 2 mm and allowed to achieve complete hardening for 7 days. Afterward, the samples were released from the molds and crushed into powders using a mortar and pestle. The samples were tested using FTIR to identify the chemical functional groups of the cement materials and by XRD to identify their phases and crystalline structures.

### Assessment of Sealer Flow

Based on ISO 6876/2012, each sealer was mixed, and using a graduated pipette, 0.05 mL of the material was dropped onto a glass plate measuring 40 × 40 mm and 5 mm in thickness. At 180 ± 5 seconds, after the beginning of mixing, a second glass plate was placed centrally on top of the sealer, followed by a 100-g weight ( Supplementary Fig. S1 ; available in the online version). At 10 minutes, after the beginning of mixing, the weight was removed and the maximum and minimum diameters of the compressed disc of sealer were measured. If the diameter measurements agreed to within 1 mm, the mean of both diameters was recorded. If both diameters were not within 1 mm, the test was repeated. The procedure was repeated twice more for a total of three determinations. The mean value of the three measurements was calculated to the nearest millimeter. Five specimens were used from each material prepared from five different mixtures.

### Assessment of Film Thickness

Based on ISO 6876/2012 for the film thickness test, the combined thickness of two glass plates, each measuring 5 mm in thickness and having a surface area of 200 mm ^2^ , was measured with a micrometer to an accuracy of 1 mm. The materials were mixed and placed on one glass plate, and the other plate was placed over the sealer and inserted into a loading device (Universal Testing Machine, GOTECH AI-7000-S, Taiwan). A load of 150 N was applied until the sealer filled the area between the glass plates. Ten minutes after the start of mixing, the thickness of the combined glass plates and sealer was measured using a micrometer ( Supplementary Fig. S2 ; available in the online version only). Five specimens were used from each material prepared from five different mixtures.

### Assessment of Cytotoxicity

A total of 100 mL of 3T3 mouse embryo fibroblast cell suspension was grown at 37°C in a humidified atmosphere with 5% CO _2_ in 96-well plates at a density of 3,000 cells/well for 24 hours. The sealer extracts were added to the cells at 1:1 and 1:2 dilutions (100 mL/well) using the growth medium as the dilution material. Three replications were run for each dilution. The cells were incubated at 37°C, 5% CO _2_ . Cell viability and proliferation were measured at 1, 3, and 5 days of incubation by adding 10 mL MTT reagent (5 mg/mL) to each well and incubating at 37°C, 5% CO _2_ for 4 hours. The solubilization reagent (100 mL) was then added to each well, and the absorbance was measured at a wavelength of 450 nm using an ELISA reader (Bio-Rad Laboratories, Singapore).

## Statistical Analysis

Statistical analysis was performed on flow and film thickness. The normality of the data was tested using the Shapiro–Wilk test. Statistical analysis was performed with one-way analysis of variance (ANOVA). The level of significance was set at *p* < 0.05.

## Results

### The Fourier Transform InfraRed Analysis

**Fig. 1:**
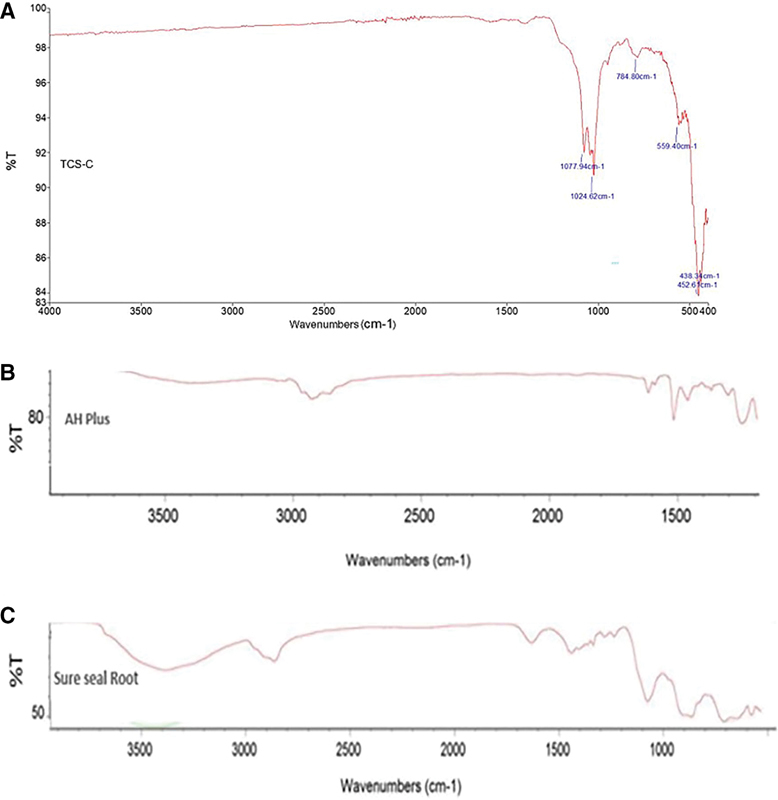
FTIR spectra after the sealers had set completely for 7 days. ( **A** ) TCS-C; ( **B** ) AH Plus; ( **C** ) Sure-Seal Root. FTIR analysis demonstrated the chemical bonding of the materials. FTIR, the Fourier Transform InfraRed; TCS-C, tricalcium silicate–chitosan.

The published literature indicates that tricalcium silicate cement has some typical FTIR peaks at 996; 938; 906; 883; 812; 524; and 464/cm. The novel TCS-C sealer showed peaks at 1,077 and 1,024/cm (absorption of silicate ion groups); 784/cm(uptake of the CH aromatic group out of the bond); and 559, 438, and 407/cm (calcium silicate fingerprint absorption). An additional peak was also evident at 407.45/cm ( Fig. 1A ) but was still considered a typical absorption of calcium silicate. The AH Plus sample showed 20 main peaks in the wavelength range of 562.60 to 802.34/cm and included the typical group absorptions (fingerprints) at 914.02/cm (absorption of –CH groups on the monosaccharide ring), 1,032.37 to 1,100.05/cm (C–O group absorption), 1,181.49 to 1,240.64/cm (asymmetric COC group absorption), 1,295.65/cm (hydroxyl group absorption), 1,361.23 to 1,508.15/cm (CN amide III group absorption), 1,581.75 to 1,606.73/cm (NH Amide II group absorption); 2,853.32 to 3,033.42/cm (–CH group absorption); and 3,368.18/cm (–OH group absorption; b). The Sure-Seal Root sample showed 12 main peaks in the wavelength range of 594.00 to 884.28/cm and included the typical group absorptions (fingerprints) at 1,090.34 (CO group absorption), 1,250.35 (asymmetric COC group absorption), 1297.65 (hydroxyl group absorption), 1,350.34 to 1,454.03 (CN amide III group absorption), 1,645.60 (NH amide II group absorption), 2,873.90 (–CH group absorption), and 3,396.08/cm (–OH group absorption; Fig. 1C ).

### X-Ray Diffraction Analysis

**Fig. 2:**
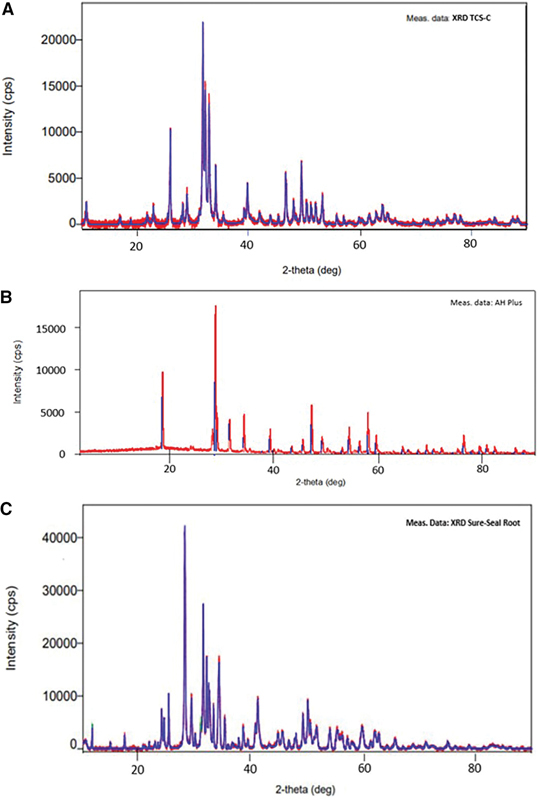
XRD results after the sealers had set completely for 7 days. ( **A** ) TCS-C; ( **B** ) AH Plus; ( **C** ) Sure-Seal Root. TCS-C, tricalcium silicate–chitosan; XRD, X-ray diffraction.

All samples showed XRD patterns with sharp and narrow peaks, indicating that the cements consisted of crystalline materials. The XRD pattern of the novel TCS-C ( Fig. 2A ) also showed similar characteristics, together with the existence of an amorphous background at 15 to 350 which probably comes from chitosan. Qualitative analysis of the XRD pattern of the novel TCS-C revealed a single hydroxyapatite phase which was confirmed by matching all its peaks to the reference (COD no. 96–900–2220). The XRD pattern of AH Plus ( Fig. 2B ) showed typical peaks for scheelite/calcium tungstate (CaWO _4_ ; ICDD no.: 00–041–1431) and baddeleyite/zirconium oxide (ZrO _2_ ; ICDD no.: 00–037–1484). These findings were in accordance with the literature which states that AH Plus contains calcium tungstate, iron oxide, and zirconium oxide. 11 13 14 The XRD pattern of Sure-Seal Root ( Fig. 2C ) indicated the presence of hatrurite/tricalcium silicate (Ca _3_ SiO _5_ ; COD no. 96–900–8367), baddeleyite/zirconium oxide (ZrO _2_ ; COD no.: 96–900–7486) and anatase/titanium dioxide (TiO _2_ ; COD no.: 96–900–9087).

### Flow

**Fig. 3:**
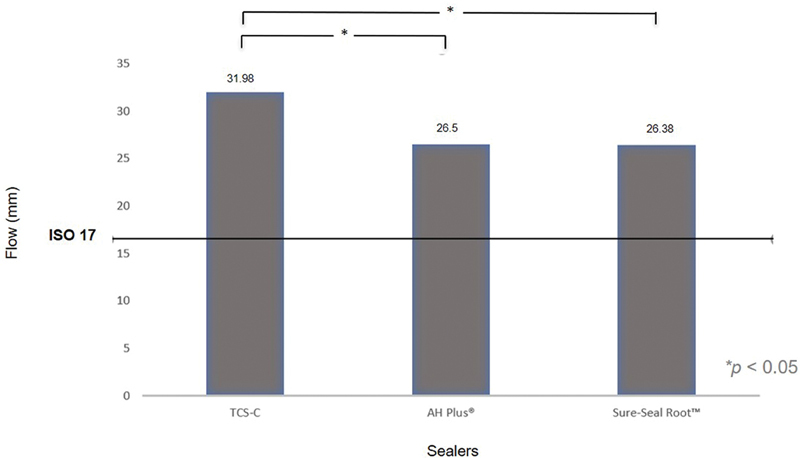
Flow ability (mm) value comparisons of TCS-C, AH Plus, Sure-Seal Root, and ISO values. The asterisk (*) shows statistically significant difference between two groups. TCS-C, tricalcium silicate–chitosan.

The novel TCS-C hybrid sealer showed a mean flow of 31.98 ± 0.68 mm, while that of Sure-Seal Root was 26.38 ± 0.69 mm and that of AH Plus was 26.50 ± 0.12 mm ( Fig. 3 ). The one-way ANOVA test showed a statistically significant difference ( *p* = 0.001) between the novel TCS-C sealer, AH Plus, and Sure-Seal Root, but no statistically significant difference ( *p* = 1.00) was detected between AH Plus and Sure-Seal Root. Measures by the examiner showed good agreement with an intraclass correlation coefficient of 0.979.

### Film Thickness

**Fig. 4:**
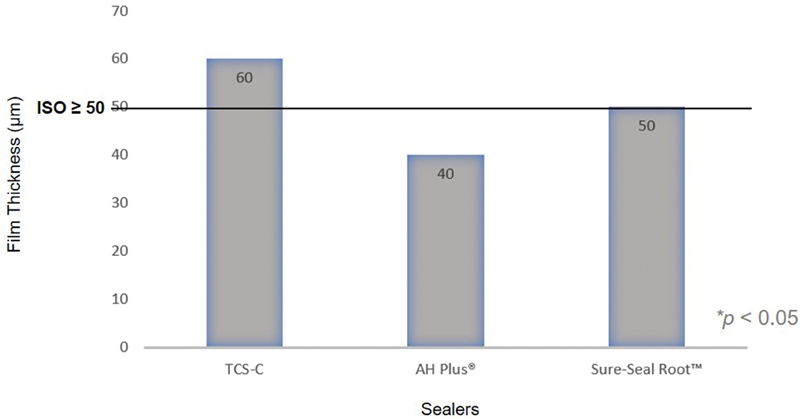
Film thickness (μm) value comparisons between TCS-C, AH Plus, Sure-Seal Root, and ISO values. TCS-C, tricalcium silicate–chitosan.

The one-way ANOVA test showed no statistically significant difference between the novel TCS-C sealer, AH Plus, and Sure-Seal Root ( *p* = 0.415). The novel TCS-C hybrid sealer showed a mean flow of 60 ± 10.0 mm, while that of Sure-Seal Root was 50 ± 10.0 mm and that of AH Plus was 40 ± 15.8 mm ( Fig. 4 ). Measures by the examiner showed good agreement with an intraclass correlation coefficient of 0.801.

### Cytotoxicity

**Fig. 5:**
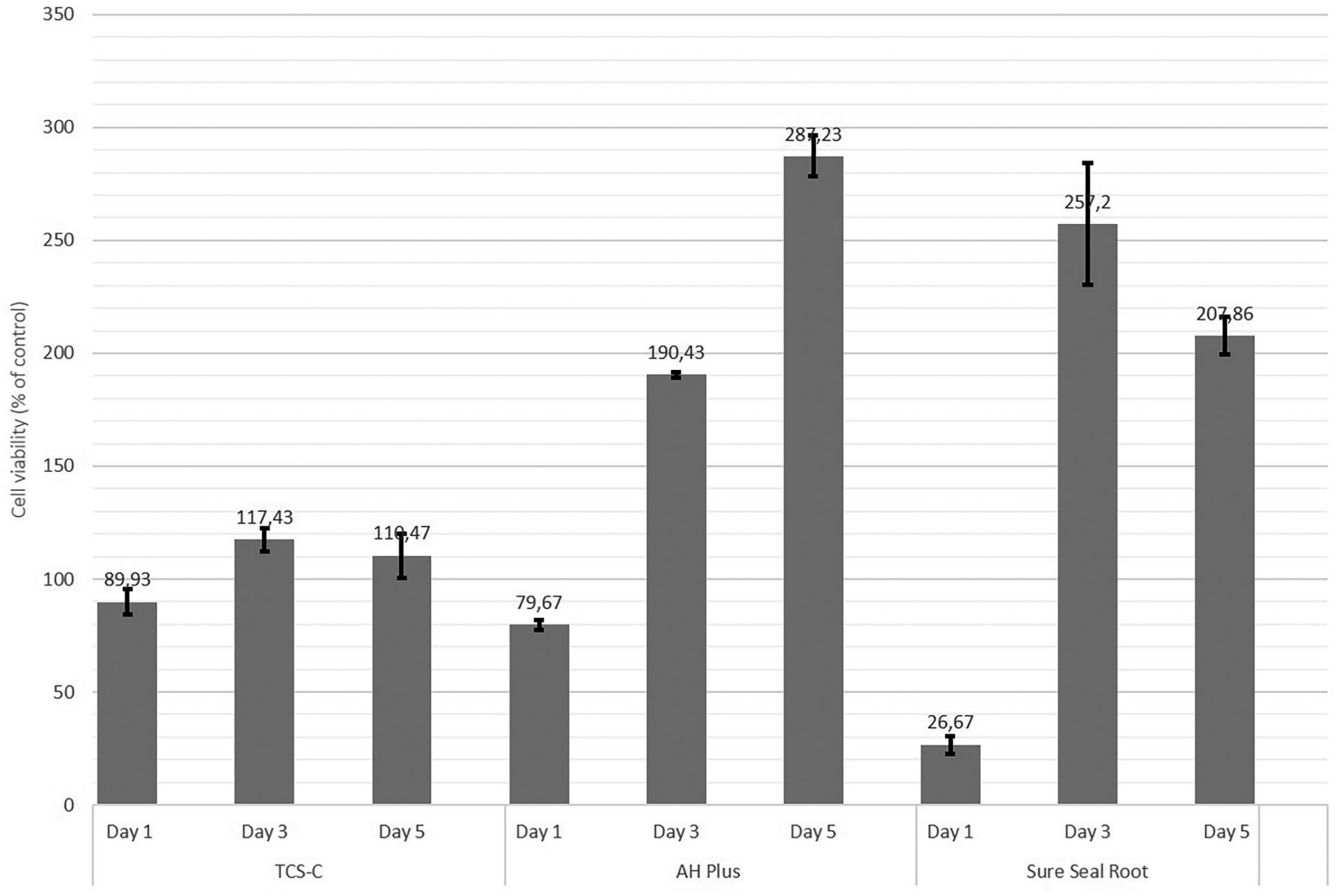
The cell viability after application of the sealers at 1:1 dilution for different times.

**Fig. 6:**
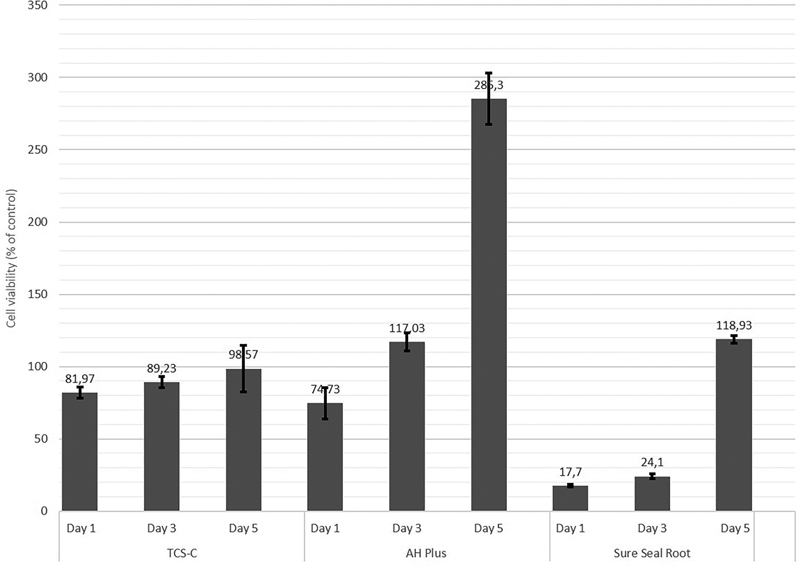
The cell viability after application of the sealers at 1:2 dilution for different times.

The cytotoxicity of the sealers was indicated by the viability of fibroblast cells compared with untreated control fibroblasts. The classification level of toxicity ( Table 1 ) was based on Dahl et al. 15 Figs. 5 and 6 show that the novel TCS-C sealer had low to no cytotoxicity at all concentrations and exposure times. By contrast, AH Plus showed low cytotoxicity at both concentrations at 1 day, and the cytotoxicity decreased with the length of exposure, whereas Sure Seal Root showed high levels of cytotoxicity at both concentrations at 1 day exposure, but its cytotoxicity also decreased with increasing exposure time. Measures by the examiner showed good agreement with an intraclass correlation coefficient of 0.934.

**Table 1:** Cytotoxicity was rated based on cell viability relative to control 15

**Interpretation**	**% of cell viability**
Noncytotoxic	Greater than 90
Slightly cytotoxic	60–90
Moderately cytotoxic	30–59
Strongly cytotoxic	Less than 30

## Discussion

### The Fourier Transform InfraRed and X-Ray Diffraction

The FTIR and XRD analyses were conducted 7 days after mixing the sealers to ensure that the three samples were perfectly set. The samples were then crushed with a mortar and pestle. The FTIR results for TCS-C showed the expected absorptions of all the ingredients of the mixture, namely, calcium silicate cement, calcium phosphate, calcium hydroxide, and chitosan ( Fig. 1A ). The XRD results indicated that the novel TCS-C sealer showed significant hydroxyapatite formation ( Fig. 1A ). Based on the literature, hydroxyapatite has a peak at 32-degree 2θ, and this was clearly seen in the samples of novel TCS-C and Sure-Seal Root. Khalil et al analyzed the properties of tricalcium silicate sealers and stated that the peaks at 32- and 34-degree 2θ were tricalcium silicate phases. 13

The addition of chitosan to tricalcium silicate cement does not change the crystalline phase of tricalcium silicate cement ( Fig. 2A ). This is according to Lin et al in his research developing hybrid calcium silicate cement by mixing different types of chitosan for bone repair materials which states that the addition of chitosan in calcium silicate material does not eliminate the crystalline phase of the material but accelerates the setting time and mechanical strength of cement. 9

The XRD results for AH Plus showed crystalline peaks indicative of calcium tungstate and zirconium oxide. Calcium tungstate is added to AH Plus as a radiopacifier. 14 16 These results are in accordance with the literature stating that AH Plus contains calcium tungstate, iron oxide, and zirconium oxide. 11 13 14 Both the AH Plus and Sure-Seal Root materials showed a zirconium oxide phase in their XRD results ( Figs. 2B and C ). Based on these characteristics, we concluded that the three cements had different material compositions; therefore, the different behaviors observed in the physical property and biocompatibility tests were expected.

### Flow

The flow ability tests were performed on the three sealers using the ISO 6876/2012 test method. The aim of this test is to evaluate the ability of the sealers to fill up the narrowest area in the root canal. In this method, the flow is described by the diameter of the sealers after imposing a load of 120 g. This load reflects the stress that sealers receive clinically in the root canal during their application with a syringe (i.e., Sure-Seal Root), with a lentulo instrument or manually with gutta-percha. The flow of the sealer also depends on its viscosity, as a low viscosity will give a higher flow. Statistical analysis of the results indicated that the TCS-C showed the highest flow ability. This was possibly due to its high water/powder (W/P) ratio; most tricalcium silicate–based sealers have W/P values of 0.3. 17 18 However, W/P values used for calcium silicate-based cement depend on its particle size. Water-soluble chitosan (2%) used in this study showed low viscosity, enough to improve cement's viscosity, and provides flow ability. Tricalcium silicate cement without chitosan addition was textured like a wet sand. Although no maximum limit is specified, the ISO standards flow rate is >17 mm ( Table 2 ). In contrast to Khalil et al, who determined an AH Plus flow rate of 17 ± 1.6 mm, this study found an AH Plus flow rate of 26.50 ± 0.12 mm which was not statistically and significantly different from that of the Sure-Seal Root at 26.38 ± 0.69 mm. 13

**Table 2:** Physicochemical properties of novel TCS-C hybrid sealer, AH Plus, and Sure-Seal Root

	**ISO standards**	**TCS-C**	**AH Plus**	**Sure-Seal Root**
Flow (mm)	>17	31.98 (0.68)	26.50 (0.12)	26.38 (0.69)
Film thickness (μm)	≤50	60 (10.0)	40 (15.8)	50 (10.0)

Abbreviation: TCS-C, tricalcium silicate–chitosan.

### Film Thickness

Based on the ISO test method, when a sealer is given a certain load, it will flow and produce a layer of a specific thickness which is related to the properties of endodontic material, namely, its shear thinning (pseudoplastic) behavior. 19 The viscosity of the material will decrease as the amount of load applied increases (followed by an increase in the shear rate). 20 Therefore, the clinician can apply excessive pressure to allow the material to enter hard-to-reach areas.

The TCS-C showed the highest film thickness value, at 60 ± 10 mm which means it has a high viscosity; in fact, its value is higher than the ISO value (≤50 µm; Table 2 ). By contrast, AH Plus at 40 ± 15.8 mm meets the ISO values, while Sure-Seal Root, at 50 ± 10, is slightly above the ISO value. Meeting the ISO-based film thickness may not be as important for tricalcium silicate–based sealers because they rely on their bioactive properties to bind to the root canal dentin. The thickness of the film does not affect the sealing ability of the sealer. 13 The high film thickness of the novel TCS-C may also be influenced by the particle size of the tricalcium silicate powder, so attempts to reduce the particle size could also reduce the film thickness.

### Cytotoxicity

The novel TCS-C sealer showed good biocompatibility, as indicated by the high fibroblast cell viability values at both concentrations and at all three exposure times ( Figs. 5 and 6 ). Conversely, AH Plus and Sure-Seal Root were both cytotoxic at both concentrations at the 1-day exposure time. However, this cytotoxicity occurred only at the initial phase, and the viability of fibroblast cells increased with the length of the exposure time. The TCS-C sealer showed stable cell viability values at various exposure times, even at the initial exposure, in contrast to most other root canal cements, which show cytotoxicity at initial exposure that diminishes over time. 21 22 These properties indicated that the novel TCS-C sealer material is not cytotoxic to fibroblast cells, possibly because the novel formula used pure material without heavy metal additives and used chitosan which is a biocompatible biopolymer. The initial inflammation probably reflected the addition of calcium hydroxide. As is widely known, the presence of calcium hydroxide might cause a superficial necrosis in the region of contact of the material with the tissue due to the increase in alkalinity. The region is subsequently repaired via the formation of hard tissue. 23 Calcium hydroxide also accelerates the tissue repair process 23 and has been shown to improve the biological properties of endodontic sealers.

Sure-Seal Root gave had the poorest fibroblast cell viability values at a concentration of 1:1 and an exposure time of 1 day. This sealer contains calcium aluminosilicate, calcium sodium phosphosilicate, zirconium oxide, and a thickening agent. Some additives contained in sealers, like radiopacifiers or thickening agents, could be cytotoxic. For example, some studies have shown that bismuth oxide negatively affects the growth and proliferation of dental pulp tissue and can also lead to pulp cell death. 24 The radiopacifier in Sure-Seal Root is zirconium oxide which is known to be inert and is only leached in minimal quantities. 24 The cytotoxic effect observed with Sure-Seal Root probably reflects some unmentioned material or the calcium hydroxide content.

In general, the cytotoxicity of the sealers depended on the dilution of the extract used, and the cytotoxicity decreased when the sealer was diluted. The dilution is also justified because when the material is in contact with the tissue, the extracellular fluids continuously eliminate the leachable compounds and their concentration progressively decreases. The viability value of AH Plus and Sure-Seal Root for the 5-day period had a value above that of TCS-C. The epoxy resin of AH Plus was considered a mutagenic substance mainly due to the addition of the amine component, although the material exhibits extremely low solubility since it is hydrophobic. 23

Chitosan is known to have good biocompatibility and a good host response which is significant in hemostasis, angiogenesis, macrophage activation, and control of fibroblast proliferation. 25 However, the characteristics of chitosan vary widely with the degree of deacetylation (DD) and the molecular weight factors also influence the adhesion and proliferation of fibroblast cells. 26 Hamilton concluded that residual ash and protein play a role in cellular-material interactions because many studies have shown that cell proliferation increases with increasing ash and decreasing protein content. 26 Whether the residual ash content is likely to be a calcium-based material is unclear, because the main component of arthropod cuticles is calcium carbonate. This is in accordance with the content of TCS-C, as its tricalcium silicate and chitosan material make it biocompatible even upon initial contact with fibroblast cells.

These findings suggest that novel TCS-C sealer is a promising bioceramic sealer that has a good biocompatibility on fibroblast cell. However, it is necessary to evaluate several other properties of the material, including setting time, sealing ability, solubility, and other physical properties, to make it an ideal endodontic sealer. In addition, the presence of chitosan combined with elements Ca/Si/P in tricalcium silicate cement is known to play important role in the function of mineralization and bioactivity of a tissue. Therefore, the bioactivity potential of novel TCS-C sealer needs to be further investigated.

## Conclusion

Adding water-soluble chitosan may improve the physical and biologic properties of tricalcium silicate cement. The novel TCS-C sealer did not fully meet the physical properties of an endodontic sealer, but it was not cytotoxic to fibroblast cells. Thus, the null hypothesis was accepted. Given its good characteristics, this sealer is considered to represent a promising bioceramic sealer in the future.
