# Supplementary material for: Characterization, Physical Properties, and Biocompatibility of Novel Tricalcium Silicate–Chitosan Endodontic Sealer
Source: Eur J Dent. 2022 Sep 5;17(1):127–35. doi: 10.1055/s-0042-1745774 (PMC9949928; doi:10.1055/s-0042-1745774)
Supplement: Supplementary file 1 — Supplementary Material [file 10-1055-s-0042-1745774-s21111852.pdf]

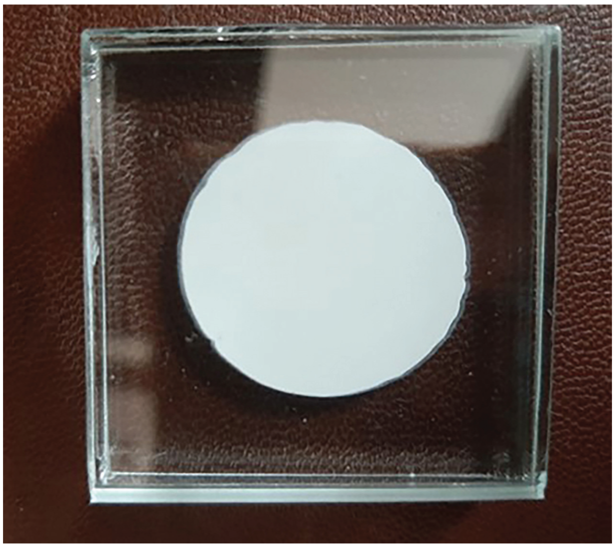

Supplementary Fig. S1 The diameter of the sample that has been pressed with another glass plate and 100 g weight is measured.

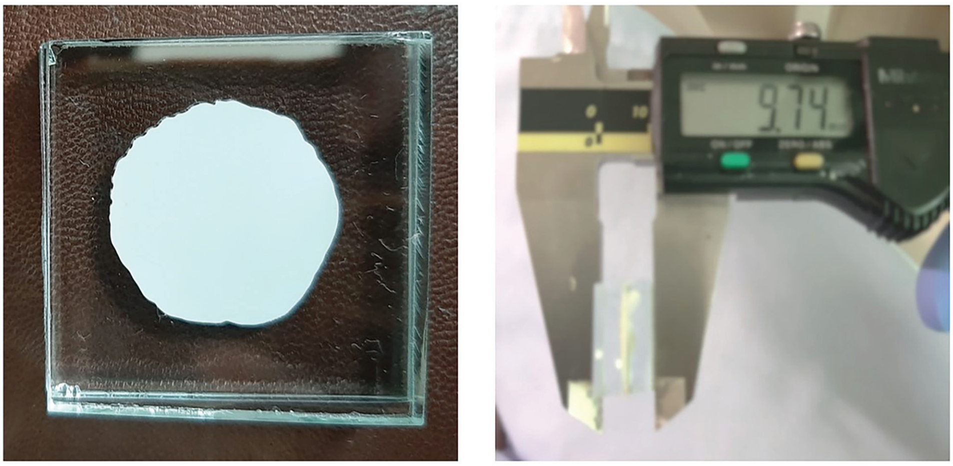

A B

Supplementary Fig. S2 The thickness of the combined glass plates and sealer after being pressed by a load of 150 N was measured using a micrometer.

 © 2022. The Author(s).
